# Obituary

**Published:** 1894-06

**Authors:** 


### Obituary.

Dr. Maitland L. Mallory, of Rochester, died at his home in that city Saturday, April 28, 1894, from an overdose of chloral, at the age of forty-six years. He had been giving much time of late to microscopic investigations, and it is believed that overwork brought on insomnia. Dr. Mallory had devoted himself especially to bacteriology and microscopic work of various kinds. He was a member of the British Royal Microscopic Society, of the American Microscopical Society and of several smaller scientific bodies. He had served for two years as a member of the Rochester Board of Health, and had particularly interested himself in the recent efforts to eradicate tuberculosis.

Dr. George W. Nesbitt, of Sycamore, Ill., died at his home April 27, 1894. Dr. Nesbitt was an alumnus of Buffalo University Medical College, class of '66. He became a leading physician in Illinois, and was prominent in the counsels of the State Medical Society, having at one time been elected its vice-president.

